# Predictive Role of Soluble B‐Cell Maturation Antigen in Short‐Term Monitoring of Differently Treated Multiple Myeloma Patients: A Prospective Study

**DOI:** 10.1002/jcla.25151

**Published:** 2025-01-16

**Authors:** Laura Caponi, Maria Livia Del Giudice, Alice Botti, Silvia Ursino, Alberto Gennari, Aldo Paolicchi, Sara Galimberti, Gabriele Buda

**Affiliations:** ^1^ Clinical Pathology Laboratory, Pisa University Hospital, Department of Translational Research and New Technologies in Medicine University of Pisa Pisa Italy; ^2^ Hematology Division, Pisa University Hospital, Department of Clinical and Experimental Medicine University of Pisa Pisa Italy

**Keywords:** BCMA, biomarker, multiple myeloma, prospective study, sBCMA

## Abstract

**Background:**

The management of multiple myeloma is challenging because the disease is incurable and unexpected relapses can threaten a patient's survival. Several assessment systems are currently available, but they often require invasive or costly procedures (e.g., instrumental bone marrow and whole‐body examinations) or rely on non‐specific markers in blood and urine that may not be sufficient to assess and monitor the disease.

**Aims:**

To address some of these limitations, the aim of this study was to evaluate the potential use of soluble B‐Cell Maturation Antigen (BCMA), a promising new serum biomarker, as a toll for moniting multiple myeloma patients.

**Materials & Methods:**

An unselected cohort of 57 newly diagnosed or relapsed myeloma patients was followed up for 6 months after starting a new therapy. Soluble BCMA levels were measured in peripheral blood using a simple and inexpensive ELISA assay.

**Results:**

Soluble BCMA was detectable in peripheral blood by a simple and inexpensive assay in all patients, even in non‐secretory disease or during BCMA‐targeted therapies, and significant changes in its levels were observed over time. The analysis showed that the decrease in sBCMA at 1 and 6 months reflects the quality of the clinical response to anti‐myeloma regimens.

**Discussion & Conclusion:**

The data provide interesting insights into the usefulness of sBCMA as a non‐invasive tool for early assessment of treatment efficacy. Its simple and cost‐effective detection in peripheral blood could provide clinicians with an addiotional resource for monitoring disease progression and tailoring treatment strategies.

## Graphical abstract

The management of multiple myeloma presents a significant challenge because of the incurability of the disease and the potential for unexpected relapses to threaten patient survival. This study evaluates the potential utility of soluble BCMA, a novel serum biomarker, in an unselected cohort of 57 newly diagnosed or relapsed myeloma patients. The presence of soluble BCMA was detectable in the peripheral blood through a simple and economical diagnostic test in all subjects. The decline in sBCMA levels is indicative of the efficacy of anti‐myeloma therapeutic strategies.

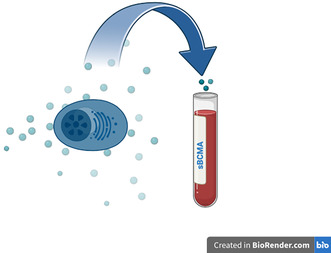

## 1 Introduction

Multiple myeloma (MM) is the second most common haematological neoplasm and it is a plasma cell dyscrasia with systemic involvement [1]. Although the availability of new therapies has significantly improved survival in recent years [2, 3], the disease can still be defined as incurable. This is why treatments with new mechanisms of action that can circumvent possible acquired drug resistance are required [4] and new biochemical markers that can serve as therapeutic targets and as tools to assess disease progression are needed. In view of the complexity of MM presentation, periodic evaluation of biomarkers in peripheral blood, together with morphological, cytofluorimetric and molecular biology reassessment in bone marrow, and whole‐body imaging investigations, is essential. All these approaches are complementary; however, laboratory biomarkers are considered to be less expensive, easier to obtain and repeatable, although they may have limitations. Historically, two main laboratory biomarkers for MM assessment have been employed: the immunoglobulin (M protein) and the free light chain, both of which are secreted by the monoclonal clone [5]. The detection and quantification of these biomarkers are essential for the diagnosis and monitoring of MM. The former is qualitatively defined by immunofixation and quantified by serum protein electrophoresis (SPEP). Nevertheless, precise quantification of M proteins when present in low quantities is challenging to achieve through SPEP, and patients with non‐secretory MM may exhibit a lack of evidence of disease with this assay. Recently, new mass spectrometry‐based methods have been strongly advocated for more effective monitoring of M protein, but they are not yet routinely used in clinical practice [6, 7, 8]. On the other hand, free light chain (FLC) assays benefit from a shorter half‐life in serum of FLC (3–6 h) as compared to the entire immunoglobulin, which more accurately reflects the rapid changes associated with the disease [9]. However, they are not useful in non‐secretory MM either, and are also strongly influenced by renal function, which is often impaired in MM patients [10]. In addition, the accuracy of immunochemical FLC tests remains a matter of debate because of the occasional discrepancies in results between different tests on the same specimen [11]. Finally, the minimal residual disease (MRD) is currently assessed by multiparametric flow cytometry or next‐generation sequencing on bone marrow samples obtained through invasive procedures. Furthermore, these techniques require a high degree of expertise, and are associated with significant costs. In addition, MRD assessment is only applicable when a satisfactory response to therapy has been achieved; it is therefore inappropriate for routine follow‐up of all patients [12]. For this reason, new biomarkers need to be defined. The aim of this paper was to investigate the potential of the circulating soluble form of B‐cell maturation antigen (sBCMA) as a biomarker for this purpose. BCMA is a membrane protein expressed by normal plasma cells; it is also highly expressed by malignant plasma cells. It is a member of the tumour necrosis factor receptor family and functions as a receptor [13]. It has been identified as a promising target for some of the most innovative and effective new therapies introduced in myeloma [14, 15, 16, 17, 18, 19, 20]. BCMA plays a critical role in the survival, clonal expansion and reconditioning of the microenvironment surrounding the tumour cells through its binding to B‐cell activation factor (BAFF) and a proliferation‐inducing ligand (APRIL) [21, 22, 23]. The BCMA extracellular portion (sBCMA) is released as a soluble peptide into the circulation because of the presence of a ubiquitous membrane γ‐secretase. Consequently, it is detectable in human blood, and its concentration is independent of renal function. In MM patients including those with non‐secretory MM, sBCMA levels have demonstrated significant preliminary correlations with disease status [24]. This study aims to collect information about sBCMA, exploring prospectively the sBCMA behaviour in an unselected population of MM patients undergoing different life‐saving treatments.

## 2 Methods

Soluble BCMA has been evaluated in newly diagnosed (transplant eligible, TE; non‐transplant eligible, nonTE) or relapsed MM patients followed up at the Hematology Division, Pisa University Hospital, and undergoing different life‐saving treatments from March 2022 to January 2024 with a laboratory assessment at the Clinical Pathology Laboratory, Pisa University Hospital, using Human BCMA/TNFRSF17 DuoSet‐ELISA, R&D Systems, MN‐USA. sBCMA levels in peripheral blood samples collected at three time points were evaluated: just before the start of treatment (T0), after 1 month (T1) and after 6 months (T2) during treatment. The percentage reduction in sBCMA was calculated as (sBCMA_T0_ ‐ sBCMA_T2_/sBCMA_T0_) × 100. For the evaluation of sBCMA, 1 mL of peripheral blood in heparin anticoagulant was collected in addition to the blood samples provided by the patient, without any modification in any standard routine evaluation. Each sample was pseudonymised. Each patient was followed up prospectively, and the disease was regularly monitored with serum and urinary markers, bone marrow and whole‐body imaging, as per standard clinical practice. The trial received approval from the local Ethics Committee (#21711) and patients provided informed consent. Quality of response was assessed by the IMWG consensus criteria [12]. Statistical analysis (descriptive statistics, Spearman correlation, Kruskal–Wallis with post hoc and Mann–Whitney tests) were performed using GraphPad Prism version 6.0 for Macintosh. Significance was set at 0.05.

## 3 Results

In total, 171 samples from 57 patients were evaluated: 23 newly diagnosed and 34 who had at least one relapse. Patient demographics are summarised in Table 1. No outliers were observed for renal impairment (i.e., eGFR< 40 mL/min per 1·73 m^2^ or creatinine > 2 mg/dL). sBCMA was measurable in all patients, including those with solitary plasmacytoma and non‐secretory disease (2 patients) and those receiving anti‐BCMA‐targeted therapies (5 patients, treated with belantamab mafodotin and teclistamab); further details on the treatments received are provided in Table 2. The sBCMA levels showed extensive diversity at T0: median sBCMA values at T0 were higher in newly diagnosed patients than in relapsed patients. Value distribution was normalised by log transformation and statistical analysis revealed a significant difference in sBCMA log at T0 both across five subgroups (one‐way ANOVA *p* = 0.0009) and on patients grouped in two categories at diagnosis (TE or nonTE) and at relapse (patients at first, second, third or more relapse: R1, R2 or R3, respectively) (*t*‐test *p* = 0.0335). Detailed analysis is reported in Appendix S1: (1A–1F).

**TABLE 1:** Patients' baseline characteristics.

Age—year median (range: min–max; IQR)	69 (53–87; IQR 13)
Gender: female/male, *n* (%)	21 (36.8)/36 (63.2)
ECOG	
0	8 (14.1)
1	28 (49.1)
2	15 (26.3)
3	6 (10.5)
M Protein	
IgG κ	26 (45.6)
IgG λ	5 (8.7)
IgA κ	7 (12.3)
IgA λ	5 (8.7)
Light chain κ or λ	5 (8.7) / 4 (7.1)
IgD λ or IgM κ	1 (1.8) / 1 (1.8)
IgG κ + IgM κ	1 (1.8)
NS/SP	2 (3.5)
Newly diagnosed pts.	23 (40.4)
ASCT eligible	10 (17.5)
Relapsed or refractory pts.	34 (59.6)
1st relapse	18 (31.5)
2nd relapse	9 (15.8)
3rd or higher relapse	7 (12.3)
Renal impairment at T0	7 (12.3)

Abbreviations: ASCT, autologous stem cell transplant; ECOG, Eastern Cooperative Oncology Group Performance Status; IQR, interquartile range; Max, maximum value; Min, minimum value; NS, non‐secretory disease; Pts, patients; SP, solitary plasmacytoma disease; T0, first analysis point, before the start of treatment.

**TABLE 2:** Summary of the different treatments received by patients during data analysis.

Regimens	Patient no.	NDMM/RRMM
Belantamab mafodotin	3	0/3
DaraRD	22	8/14
Daratumumab	1	0/1
DaraVD	2	0/2
DaraVTD	11	10/1
EloPD	2	0/2
IsaKD	1	0/1
IsaPD	1	0/1
IxaRD	1	0/1
KD56	2	0/2
KRD	1	0/1
MP	1	1/0
PACE	1	0/1
PVD	1	0/1
RD	3	2/1
Teclistamab	2	0/2
VMP	2	2/0

Abbreviations: DaraRD, daratumumab plus lenalidomide‐dexamethasone; DaraVD, daratumumab plus bortezomib‐dexamethasone; DaraVTD, daratumumab plus bortezomib, thalidomide‐dexamethasone; EloPD, elotuzumab pomalidomide‐dexamethasone; IsaKD, isatuximab plus carfilzomib‐dexamethasone; IsaPD, isatuximab plus pomalidomide‐dexamethasone; IxaRD, ixazomib plus lenalidomide‐dexamethasone; KD56, carfilzomib plus dexamethasone; KRD carfilzomib plus lenalidomide‐dexamethasone; MP, melphalan plus prednisone; NDMM, Newly Diagnosed Multiple Myeloma; PACE, cisplatin plus doxorubicin‐cyclophosphamide‐etoposide; PVD, pomalidomide plus bortezomib‐dexamethasone; RD, lenalidomide plus dexamethasone; RRMM, relapsed/refractory multiple myeloma; VMP, bortezomib plus melphalan‐prednisone.

**FIGURE 1:**
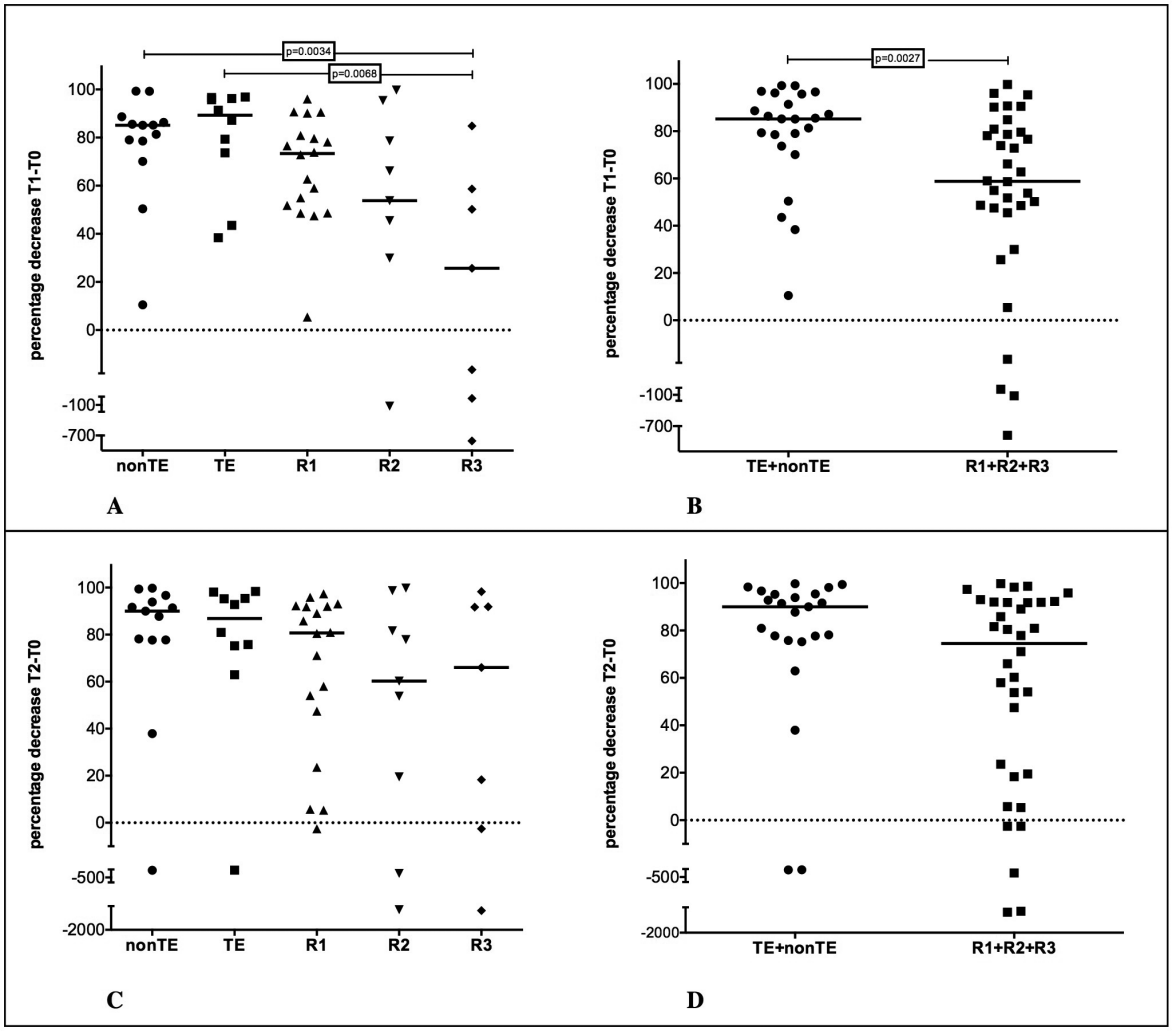
Upper graphs. (A) BCMA percentage decrease after 1 months of treatment (T1‐T0) in five patient groups with overall significance by Kruskal–Wallis with *p* = 0.0117, Mann–Whitney test: NonTE versus R3 *p* = 0.0034; Mann–Whitney test: TE versus R3 *p* = 0.0068. (B) BCMA percentage decrease after 1 months of treatment (T1‐T0) in patients grouped as newly diagnosed patients (TE + nonTE) and relapsed patients (R1 + R2 + R3) Mann–Whitney test: Newly diagnosed (TE + nonTE) versus relapsed (R1 + R2 + R3) *p* = 0.0027. Lower graphs. (C) BCMA percentage decrease after 6 months of treatment (T2‐TO) in five patient groups. (D) BCMA percentage decrease after 6 months of treatment (T2‐TO) in patients grouped as newly diagnosed patients (TE + nonTE) and relapsed patients (R1 + R2 + R3) (1D). No statistically significant difference between the groups was found. See text for details. NonTE, non‐transplant eligible; R1 patients; R1, patients at first relapse; R2, patients at second relapse; R3, patients at third or more relapse; TE, transplant eligible.

The percentage decrease in sBCMA after the first month of therapy (T1‐T0) was analysed both in five subgroups and in patients grouped as newly diagnosed or relapsed (data detailed in Appendix S2: 2A,B). The analysis, summarised in the upper part of Figure 1, showed moderate differences within the five groups (*p* = 0.0117) confirmed by the difference between the low median of the R3 patients and nonTE and TE (Figure 1A, see legend). When patients were grouped (newly diagnosed vs. relapsed), the median reduction was higher for the former, and the statistically significant difference was confirmed with a *p* = 0.0027 (Figure 1B). A similar analysis was performed on the percentage of sBCMA decrease after 6 months of therapy (T2‐T0) summarised in the lower part of Figure 1 (data detailed in Appendix S2: 2C,D). The percentage decrease in sBCMA from T0 to T2 was analysed again both in five subgroups (Figure 1C) and in patients grouped as newly diagnosed or relapsed (Figure 1D), in both cases without showing a statistically significant difference between the groups, even if, in the first‐line therapy, the median decrease was higher (90%) than in the salvage therapy (74.5%) and a moderate trend was observed. It is worth noting that the lack of significance at 6 months is mainly because of the only two negative sBCMA decreases occurring in the two newly diagnosed patients who experienced PD (data not shown). This may suggest that a larger number of patients may reveal statistical significance.

Quality of clinical response (QoR) was assessed after 1 and 6 months. After 6 months of therapy, 21 of the 57 patients had a complete or very good partial response (CR/VGPR), 27 had a partial response (PR) and 9 experienced progressive disease (PD). A possible relationship between the absolute levels of sBCMA (or their log) at T0 and QoR was sought, but no correlation was found, demonstrating that the therapeutic response does not depend on the sBCMA level at the start of therapy.

**FIGURE 2:**
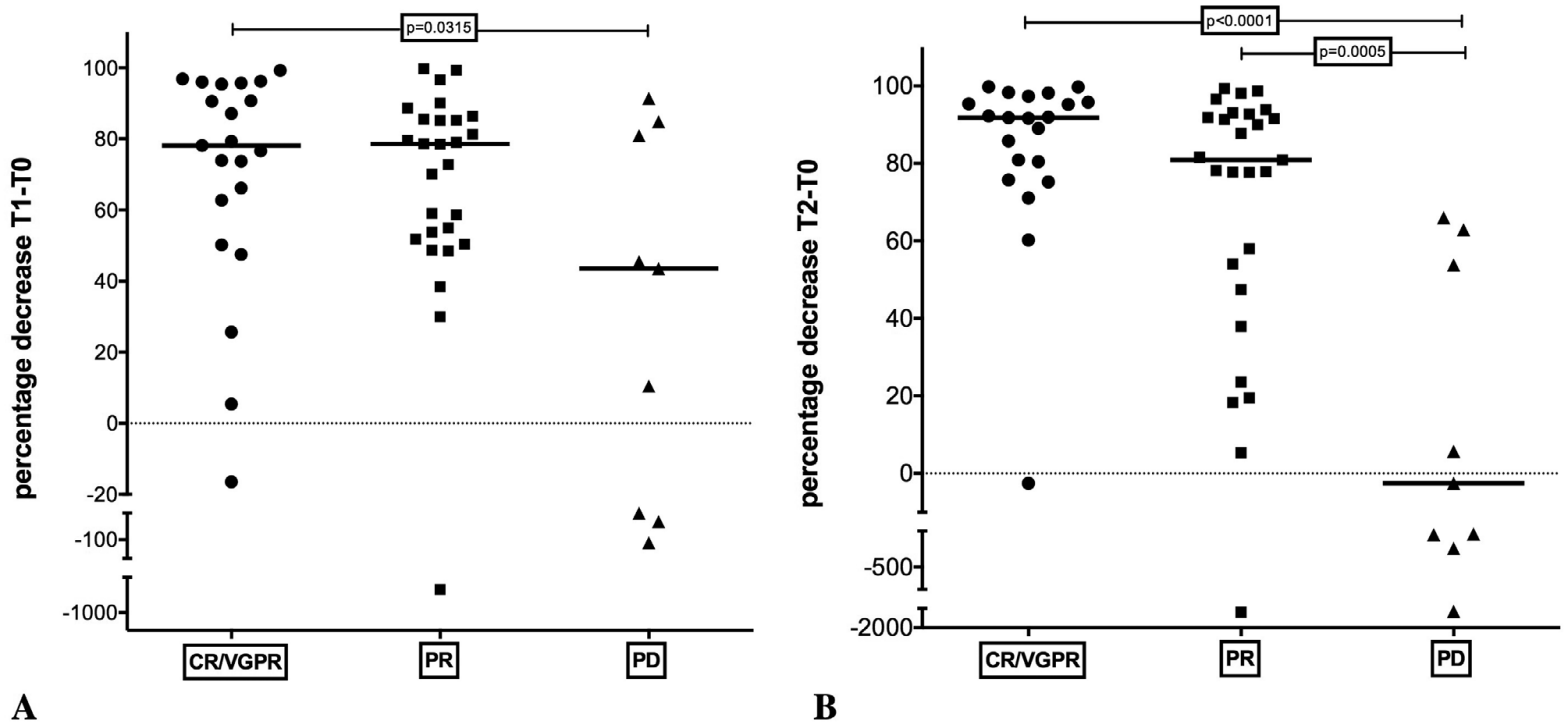
sBCMA percentage decrease after 1 month (A) and after 6 months (B) of treatment in relation to the clinical response. (A) A weak correlation was found between percentage decrease in sBCMA after 1 months (Spearman's *r* = −0.2648; *p* = 0.0465) and QoR; the Kruskal–Wallis test did not reach significance. The only significance (*p* = 0.0315) was reached between CG/VGRP and PD groups, analysing the group pairs separately (*p* = 0.0315). (B) Higher correlation was found between percentage decrease in sBCMA after 6 months (Spearman's *r* = −0.5236; *p* < 0.0001) and QoR. Kruskal–Wallis revealed significant differences among the three groups (*p* = 0.0001). Post hoc test analysis showed significant differences between the PD group and the other two, with *p* < 0.0001 and *p* = 0.0005 for the CR/VGPR or PR versus PD group, respectively. CR/VGPR, complete response/very good partial response; PR, partial response; PD, progressive disease.

The QoR weakly correlated with the percentage decrease of sBCMA after 1 month (Spearman's *r* = −0.2648; *p* = 0.0465). The median decreases were 78.1%, 78.5% and 43.5% in patients with CR/VGPR, PR and PD, respectively, but the Kruskal–Wallis test on the three groups did not reach statistical significance possibly because of the high variability of the data, and the only faintly significant difference (*p* = 0.0315) appeared between patients with CR/VGPR and those with PD when analysed separately by the Mann–Whitney test (Figure 2A).

When the percentage decrease in sBCMA after 6 months was evaluated in relation to response, a higher statistically significant correlation was found (Spearman's *r* = −0.5236; *p* < 0.0001). In CR/VGPR patients, the median T2–T0 was 91.8%, whereas in PR and PD it was 80.9% and −2.5%, respectively. Further analysis by Kruskal–Wallis showed a highly significant difference (*p* = 0.0001). Post hoc analysis (Dunn) followed by Mann–Whitney did not show significant differences between the decrease in the CR/VGPR and PR groups (although a trend seemed to appear with *p* = 0.0737), whereas it demonstrated a highly statistically significant difference between PD patients and the other two groups (CR/VGPR and PR), showing *p* < 0.0001 and *p* = 0.0005, respectively (Figure 2B). Data are detailed in Appendix S3: (3A–B).

## 4 Discussion

Multiple myeloma requires constant efforts to identify new disease monitoring tools that can predict relapses and allow tailoring of pharmacological treatment strategies. This study focuses on the potential utilisation of sBCMA as a novel useful biomarker in MM management alongside the established monitoring tools in myeloma patients. Circulating B‐cell maturation antigen (sBCMA) is released in the peripheral blood of patients with active multiple myeloma, but this protein can also be measured in healthy subjects [25, 26]. It has already been studied in patients with asymptomatic monoclonal gammopathies as a potential tool for stratifying patients at risk of progression to symptomatic disease [27, 28]. In this study, sBCMA was evaluated in an heterogeneous population of MM patients with active multiple myeloma requiring systemic therapy. It was measurable in each patient at each study time point in blood samples collected before initiation and during treatment administration, with a reproducible and inexpensive technique. It was also detectable in patients with non‐secretory disease or those treated with anti‐BCMA drugs (such as belantamab mafodotin and teclistamab), confirming what has previously been investigated during targeted therapy [29, 30]. The behaviour of sBCMA over time has shown that its median percentage reduction is higher in patients who achieve a response than in those who relapse. This evidence is stronger in the samples of this study when the reduction is assessed at 6 months rather than at 1 month. However, even at 1 month, the percentage decrease in sBCMA is already higher in patients who have a response at 6 months, than in those who have a progression. A greater number of patients may provide stronger evidence of the performance of sBCMA as a satisfactory early monitoring biomarker. The data also suggest that the QoR is only partially influenced by the sBCMA levels at baseline, but it is their decline that reflects the measured outcome. The utilisation of this marker, which exhibits a divergent trajectory at 1 month for patients who achieve at least a partial response at 6 months compared to those who progress, could facilitate the implementation of a more tailored therapy, thereby reducing the exposure of patients to expensive molecules and their potential toxicity. To the best of our knowledge, no studies have yet focused on a heterogeneous population with multiple myeloma for the evaluation of sBCMA as a potential new biomarker. However, recent studies have investigated sBCMA fluctuations during therapy in the context of targeted treatments, such as anti‐BCMA bispecific antibodies or chimeric antigen receptor T‐cell immunotherapy [29, 30], or have previously demonstrated that sBCMA was secreted in substantial concentrations in patients at diagnosis [26]. Our findings corroborate previous observations but suggest that sBCMA may serve as a valuable biomarker across all stages of the disease and during any kind of anti‐myeloma therapy, underlining its potential as a broad‐ranging, cost‐effective and accessible tool for use in a variety of MM contexts.

The way sBCMA declines may position it as a new potential instrument in MM with robust predictive value, even in the early stages of disease reassessment during treatment. In particular, in patients responding to treatment, it seems to be a marker of response that varies at a very early stage: It could allow future adaptation of treatment, defining early strategies of intensification or discontinuation of the regimen already established. As it does not depend on renal filtration, it also seems to be an easily manageable marker. In addition, it would allow simple and non‐invasive peripheral blood monitoring at all stages of the disease, even and especially in patients with non‐secretory disease. On the one hand, the strength of this study is the inclusion of unselected patients treated with different therapeutic regimens, including some of the latest commercially available agents, such as targeted ones, and the prospective design. On the other hand, the limitations of this study are the single‐centre experience and the still limited number of patients analysed. The analysis will be expanded to include a larger cohort of patients, and other well‐known disease markers will be included in the evaluation. However, preliminary data seem extremely interesting: the way sBCMA declines makes it a potential marker with strong predictive value, even at very early stages of disease reassessment during therapy. The analysis will be extended in future to a larger cohort of patients and evaluated with the trend of well‐known disease markers, providing interesting insights into the management of MM and other plasma cell disorders.

## Author Contributions

G.B. and L.C. designed the research. L.C., A.B., S.U., A.G. and M.L.D.G. carried out the experiment. M.L.D.G., L.C., G.B., S.G. and A.P. analysed the data. M.L.D.G., L.C. and G.B. wrote the paper.

## Conflicts of Interest

The authors declare no conflicts of interest.

## Supporting information


Appendix S1.

## Data Availability

The raw data that support the findings of this study are available from the corresponding author, M.L.D.G., upon reasonable request.
