# Supplementary material for: Predictive Role of Soluble B‐Cell Maturation Antigen in Short‐Term Monitoring of Differently Treated Multiple Myeloma Patients: A Prospective Study
Source: J Clin Lab Anal. 2025 Jan 16;39(4):e25151. doi: 10.1002/jcla.25151 (PMC11848191; doi:10.1002/jcla.25151)
Supplement: Supplementary file 1 — Appendix S1. [file JCLA-39-e25151-s001.docx]

Supplementary Information (SI)

**S1 Descriptive and statistical analysis of sBCMA levels**

S1 1A Summary of the sBCMA levels (ng/ml) at T0 per patients category: non Transplant Eligible newly diagnosed patients (NonTE), Transplant Eligible newly diagnosed patients (TE), patients at first relapse (R1), at second relapse (R2), at third or more relapse (R3).

|  | sBCMA  NonTE | sBCMA T0  TE | sBCMA T0  R1 | sBCMA T0  R2 | sBCMA T0  R3 |
| --- | --- | --- | --- | --- | --- |
| Patients no. | 13 | 10 | 18 | 9 | 7 |
| Min | 44.34 | 25.71 | 9.727 | 11.53 | 40.83 |
| Max | 3041 | 512.0 | 233.1 | 1188 | 1026 |
| Median (Q_2_) | 468.7 | 108.8 | 67.12 | 127.1 | 281.9 |
| Q_1_ - Q_3_ | 166.9 - 629.5 | 31.38 - 271.6 | 32.3 - 176.5 | 70.55 - 354.5 | 147.7 - 443.5 |

The distribution of raw data was not normal. Thus median and interquartile ranges are reported.

No.: Number. Min: Minimum sBCMA Level. Max: Maximum sBCMA Level. Median (Q_2_): Median sBCMA Level. Q_1_ - Q_3_ interquartile range

S1 1B Summary of the sBCMA log levels at T0 per patients category: non Transplant Eligible newly diagnosed patients (NonTE), Transplant Eligible newly diagnosed patients (TE), patients at first relapse (R1), at second relapse (R2), at third or more relapse (R3).

|  | sBCMA  NonTE | sBCMA T0  TE | sBCMA T0  R1 | sBCMA T0  R2 | sBCMA T0  R3 |
| --- | --- | --- | --- | --- | --- |
| Patients no. | 13 | 10 | 18 | 9 | 7 |
| Min | 1.647 | 1.410 | 0.9880 | 1.062 | 1.611 |
| Max | 3.483 | 2.709 | 2.367 | 3.075 | 3.011 |
| Mean | 2.559 | 2.013 | 1.788 | 2.159 | 2.375 |
| CI interval | 2.278 - 2.840 | 1.663 - 2.363 | 1.564 - 2.013 | 1.717 - 2.601 | 1.974 - 2.776 |

The logarithmic transformation of the data produced a normal distribution. Thus mean and 95% CI interval instead of median and interquartile range were reported.

No., Number. Min: Minimum sBCMA Level log. Mean: mean sBCMA Level log. Max, Maximum sBCMA Level log. CI interval: lower and upper 95% confidence interval of the mean.

S1 1C Graphic distribution of the sBCMA log levels at T0 per patients category: non Transplant Eligible newly diagnosed patients (NonTE), Transplant Eligible newly diagnosed patients (TE), patients at first relapse (R1), at second relapse (R2), at third or more relapse (R3).


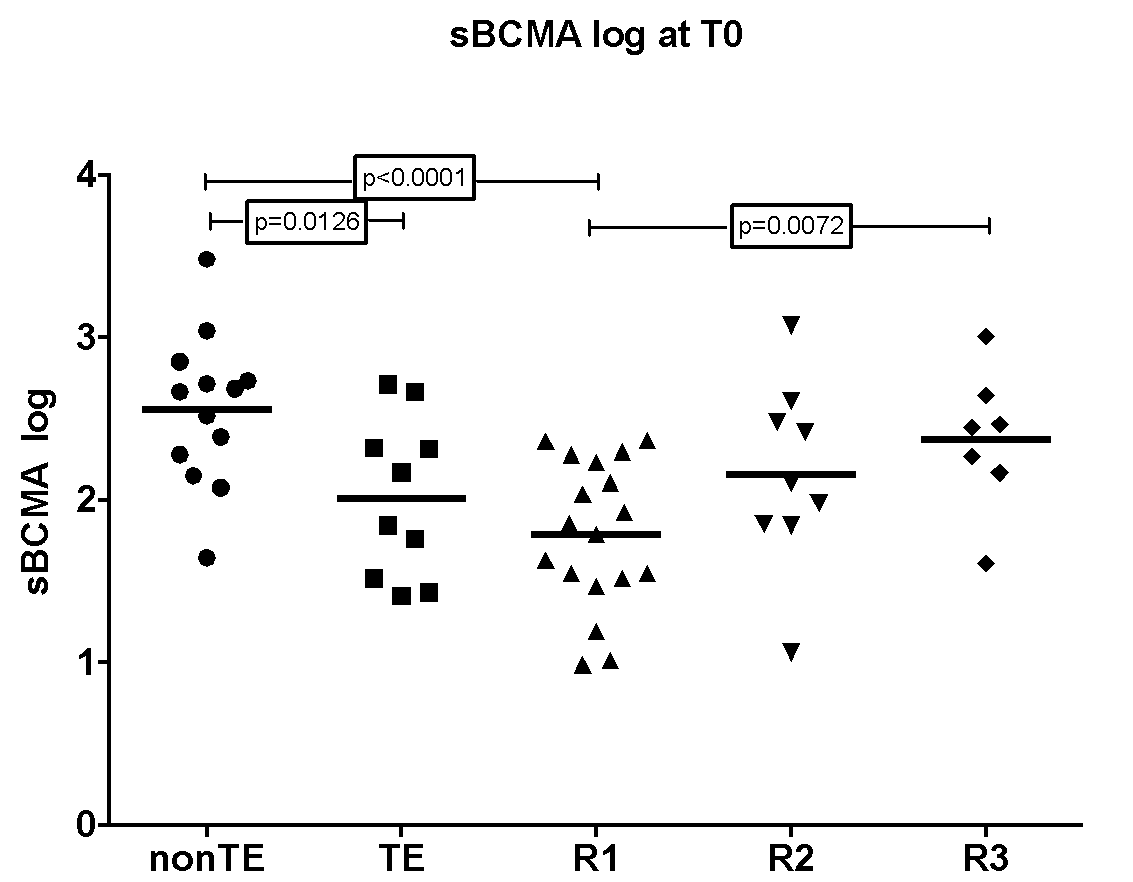


One way ANOVA analysis showed statistically significant differences between groups **(p=0.0009)**, confirmed by comparisons operated by t test with p values, as reported in Figure

S1 1D Summary of the sBCMA levels (ng/ml) at T0 in patients grouped by category: newly diagnosed (NonTE+TE) or relapsed (R1+R2+R3) patients.

|  | **sBCMA T0  nonTE+TE** | **sBCMA T0  R1+R2+R3** |
| --- | --- | --- |
| **Patients no.** | 23 | 34 |
| **Min** | 25.71 | 9.727 |
| **Max** | 3041 | 1188 |
| **Median (Q_2_)** | 209.0 | 118.0 |
| **Q_1_ - Q_3_** | 69.69 - 512.00 | 39.54 - 240.4 |

The distribution of raw data was not normal. Thus median and interquartile ranges are reported.

No.: Number. Min: Minimum sBCMA Level. Max: Maximum sBCMA Level. Median (Q_2_): Median sBCMA Level. Q_1_ - Q_3_ interquartile range

S1 1E Summary of the sBCMA log levels at T0 in patients grouped by category: newly diagnosed (NonTE+TE) or relapsed (R1+R2+R3) patients.

|  | **sBCMA T0  nonTE+TE** | **sBCMA T0  R1+R2+R3** |
| --- | --- | --- |
| **Patients no.** | 23 | 34 |
| **Min** | 1.410 | 0.9880 |
| **Max** | 3.483 | 3.075 |
| **Mean** | 2.322 | 2.007 |
| **CI interval** | 2.088 - 2.556 | 1.822 - 2.192 |

The logarithmic transformation of the data produced a normal distribution. Thus mean and 95% CI interval instead of median and interquartile range were reported.

No., Number. Min: Minimum sBCMA Level log. Mean: mean sBCMA Level log. Max, Maximum sBCMA Level log. CI interval: lower and upper 95% confidence interval of the mean.

S1 1F Graphic distribution of the sBCMA log levels at T0 per patients category: newly diagnosed (NonTE+TE) or relapsed (R1+R2+R3) patients.
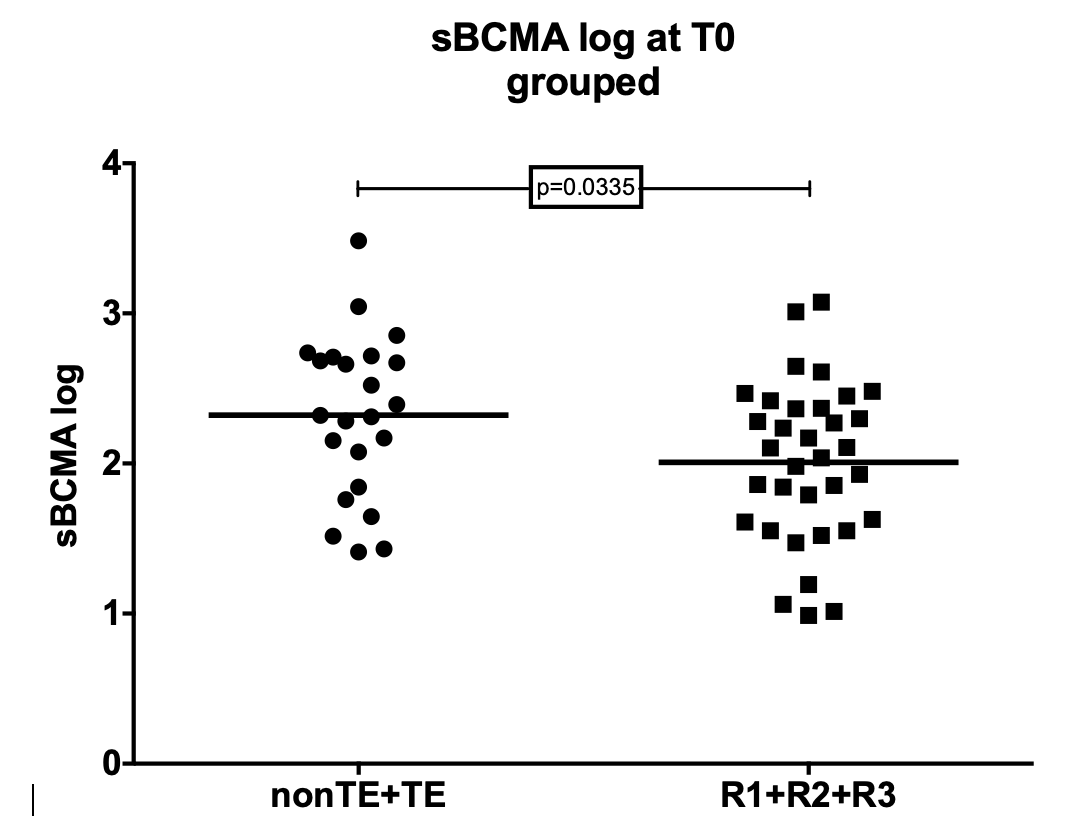


When patients where grouped by category, t test showed a statistically significant difference with a **p=0.0335**

**S2 Descriptive and statistical analysis of sBCMA percentage decrease in the different categories of patients**

S2 2A Summary of the percentage decrease of sBCMA levels after 1 months of therapy (T1-T0) per patients category: non Transplant Eligible newly diagnosed patients (NonTE), Transplant Eligible newly diagnosed patients (TE), patients at first relapse (R1), at second relapse (R2), at third or more relapse (R3).

|  | **NonTE** | **TE** | **R1** | **R2** | **R3** |
| --- | --- | --- | --- | --- | --- |
| **Patients no.** | 13 | 10 | 18 | 9 | 7 |
| **Min** | 10.49 | 38.38 | 5.409 | -108.4 | -736.0 |
| **Max** | 99.29 | 96.88 | 96.02 | 99.76 | 84.85 |
| **Median (Q_2_)** | 85.13 | 89.26 | 73.38 | 53.78 | 25.66 |
| **Q_1_ -Q_3_** | 74.33 - 87.50 | 66.16 - 96.32 | 51.01 - 83.21 | -0.09 - 87.03 | -52.56 - 58.63 |

The distribution of raw data was not normal. Thus median and interquartile ranges are reported.

Kruskal-Wallis test: p=0.0117; Mann Whitney test: nonTE vs R3 p=0.0034; Mann Whitney test: TE vs R3 p=0.0068

No.: Number. Min: Minimum percentage sBCMA decrease. Max: Maximum percentage sBCMA decrease. Median (Q_2_): Median percentage sBCMA decrease. Q_1_ - Q_3_ interquartile range

S2 2B Summary of the percentage decrease of sBCMA levels after 1 months of therapy (T1-T0) in patients grouped as newly diagnosed patients: (NonTE+TE), and patients relapsed (R1+R2+R3).

|  | **NonTE+TE** | **R1+R2+R3** |
| --- | --- | --- |
| **Patients no.** | 23 | 34 |
| **Min** | 10.49 | -736.0 |
| **Max** | 99.29 | 99.76 |
| **Median (Q_2_)** | 85.19 | 58.82 |
| **Q_1_ -Q_3_** | 73.70 - 95.71 | 41.60 - 79.95 |

Mann Whitney test: newly diagnosed (TE+nonTE) vs relapsed (R1+R2+R3) **p=0.0027**

No.: Number. Min: Minimum percentage sBCMA decrease. Max: Maximum percentage sBCMA decrease. Median (Q_2_): Median percentage sBCMA decrease. Q_1_ - Q_3_ interquartile range

S2 2C Summary of the percentage decrease of sBCMA levels (ng/ml) after 6 months of therapy (T2-T0) per patients category: non Transplant Eligible newly diagnosed patients (NonTE), Transplant Eligible newly diagnosed patients (TE), patients at first relapse (R1), at second relapse (R2), at third or more relapse (R3).

|  | **NonTE** | **TE** | **R1** | **R2** | **R3** |
| --- | --- | --- | --- | --- | --- |
| **Patients no.** | 13 | 10 | 18 | 9 | 7 |
| **Min** | -139.1 | -132.0 | -2.530 | -1575 | -1595 |
| **Max** | 99.73 | 98.37 | 97.37 | 99.77 | 98.23 |
| **Median (Q_2_)** | 90.00 | 86.85 | 80.70 | 60.26 | 66.00 |
| **Q_1_ -Q_3_** | 77.71 - 95.26 | 72.15 - 96.08 | 41.51 - 92.05 | -137.2 - 90.13 | -2.53 - 91.83 |

Kruskal-Wallis test: no statistically significant evidence was observed

No.: Number. Min: Minimum percentage sBCMA decrease. Max: Maximum percentage sBCMA decrease. Median (Q_2_): Median percentage sBCMA decrease. Q_1_ - Q_3_ interquartile range

S2 2D Summary of the percentage decrease of sBCMA levels (ng/ml) after 6 months of therapy (T2-T0) in patients grouped as newly diagnosed patients: (NonTE+TE), and patients relapsed (R1+R2+R3).

|  | **NonTE+TE** | **R1+R2+R3** |
| --- | --- | --- |
| **Patients no.** | 23 | 34 |
| **Min** | -139.1 | -1595 |
| **Max** | 99.73 | 99.77 |
| **Median (Q_2_)** | 90.00 | 74.49 |
| **Q_1_ -Q_3_** | 75.78 - 95.40 | 19.19 - 91.88 |

Mann Whitney test: no statistically significant evidence was observed (p=0.0721)

No.: Number. Min: Minimum percentage sBCMA decrease. Max: Maximum percentage sBCMA decrease. Median (Q_2_): Median percentage sBCMA decrease. Q_1_ - Q_3_ interquartile range

**S3 Descriptive and statistical analysis of sBCMA percentage decrease in patients grouped by Quality of Response**

S3 3A Summary of the sBCMA percentage decrease after 1 month of treatment (T1-T0) in relationship with the clinical response

|  | CR/VGPR | PR | PD |
| --- | --- | --- | --- |
| No. | 21 | 27 | 9 |
| Median (Q2) | 78.15 | 78.56 | 43.52 |
| Q1 - Q3 | 56.49 – 95.56 | 51.8 – 99.76 | -41.34 – 82.87 |

Median and interquartile ranges are reported. Kruskall Wallis test did not reach significance. When analysed separately with Mann-Whitney a significative difference emerged (0.0315) only between CR/VGPR and PD groups.

No.: Number. Median (Q2): Median sBCMA Level. Q1 - Q3 interquartile range CR Complete Response; VGPR Very Good Partial Response; PR Partial Response; PD Progression Disease

S3 3B Summary of the sBCMA percentage decrease after 6 months of treatment (T2-T0) in relationship with the clinical response

|  | CR/VGPR | PR | PD |
| --- | --- | --- | --- |
| No. | 21 | 27 | 9 |
| Median (Q2) | 91.83 | 80.93 | -2.533 |
| Q1 - Q3 | 78.12 – 96.61 | 47.47 – 92.77 | -216.5 – 58.36 |

Median and interquartile ranges are reported. Kruskall Wallis test reached significance p = 0.0001. When analysed separately with Mann-Whitney a significative difference emerged between PD group and CR/VGPR (p < 0.0001) and PR (p = 0.0005) respectively.

No.: Number. Median (Q2): Median sBCMA Level. Q1 - Q3 interquartile range CR Complete Response; VGPR Very Good Partial Response; PR Partial Response; PD Progression Disease
